# Magnetic Resonance Imaging: Time-Dependent Wetting and Swelling Behavior of an Auxetic Hydrogel Based on Natural Polymers

**DOI:** 10.3390/polym14225023

**Published:** 2022-11-19

**Authors:** Sandra Haas, Barbara Schmieg, Paul Wendling, Gisela Guthausen, Jürgen Hubbuch

**Affiliations:** 1 Institute of Process Engineering in Life Sciences, Section IV: Molecular Separation Engineering, Karlsruhe Institute of Technology (KIT), Fritz-Haber-Weg 2, 76131 Karlsruhe, Germany; 2 Institute of Functional Interfaces, Karlsruhe Institute of Technology (KIT), Hermann-von-Helmholtz-Platz 1, 76344 Eggenstein-Leopoldshafen, Germany; 3 Institute of Mechanical Process Engineering and Mechanics, Karlsruhe Institute of Technology (KIT), Adenauerring 20b, 76131 Karlsruhe, Germany; 4 Engler Bunte Institute Water Chemistry and Technology, Karlsruhe Institute of Technology (KIT), Adenauerring 20b, 76131 Karlsruhe, Germany

**Keywords:** magnetic resonance imaging, protein-based hydrogels, 3D printing, auxetics, localized anisotropic swelling, stimuli-responsive material

## Abstract

A time-dependent understanding of swelling characteristics and external stimuli behavior is crucial for the development and understanding of functional hydrogels. Magnetic resonance imaging (MRI) offers the opportunity to study three-dimensional (3D) soft materials nondestructively. This technique is already widely used as an image-based medical diagnostic tool and is applied here to evaluate complex structures of a hydrogel—a double network of chemically crosslinked casein enhanced with alginate—fabricated by 3D printing. When hydrogel disks immersed in four different liquid systems were analyzed, the material exhibited distinct system-dependent behavior characterized by rheological and mechanical measurements. Further material functionalization was achieved by macroscopic structuring of the hydrogel as an auxetic material based on a re-entrant honeycomb structure. MRI offers the advantage of monitoring overall changes in the area of the analyzed specimen and internal structural changes simultaneously. To assess the behavior of this complex structure, a series of short MRI measurements, each lasting 1.7 min, captured liquid diffusion and thus structural swelling behavior. A clear dependence of external and internal structural changes as a function of liquid properties causing these changes was observed. In conclusion, this approach might pave the way for prospective applications to monitor liquid diffusion into (e.g., vascularization) and swelling behavior of functional hydrogels.

## 1. Introduction

Three-dimensional (3D) polymeric structures with their swelling agent being water are known as hydrogels and are commonly used for biomedical applications such as targeted drug delivery, tissue engineering, and smart biosensors, as well as in 3D and four-dimensional (4D) printing [1,2,3]. Such applications introduce sensitive components such as cells or the need of complex material shapes. Macroscopic structuring (e.g., by an auxetic structure) can add a further dimension of functionalization to a material. Auxetic structures exhibit a negative Poisson’s ratio and thus, for example, react with a construction in certain spatial directions upon the application of mechanical stress. In comparison to unstructured objects of the same material, the introduction of an auxetic structure can improve mechanical properties such as shear resistance, fracture toughness, and resilience [4,5,6,7].

In general, hydrogels can be produced by physical or chemical crosslinking of a monomer source [8]. As one possibility, unmodified proteins can be used to obtain dityrosine- crosslinked hydrogels by crosslinking phenolic hydroxy groups via enzymatic, fenton-like, or photoinitiated reactions [9]. In the field of 3D printing, a common photoinitiator for dityrosine crosslinking is tris(2,2′-bipyridyl)dichlororuthenium(II) (Ru(bpy)_3_Cl_2_), which is induced by visible light in the presence of an electron acceptor such as ammonium or sodium persulfate [10,11,12,13]. Using this approach, stimuli-responsive hydrogels can be obtained [14,15]. In particular, these hydrogels change their structural and volume phase transition as a response to external stimuli [16]. To enhance the fabrication window in extrusion 3D printing, thickeners such as the linear and highly charged polysaccharide sodium alginate are widely used to increase the structural integrity of ink formulations [17,18]. Due to its high biocompatibility and ease of handling, alginate is also widely applied as an extrudable bioink hydrogel material itself [19], as in the presence of multivalent metal cations or in acid solutions, hydrogelation of sodium alginate is propagated by ionic crosslinking [20]. Thus, with additional crosslinking of sodium alginate, a so-called double-network hydrogel can be obtained.

Since water is an integral part of hydrogels, an improved understanding of swelling degree and mechanical stability should be obtained [21]. Current approaches to determine the swelling degree and water uptake mainly focus on weight or volume analysis of dried or as-prepared hydrogels in comparison to soaked hydrogel samples. Weight measurements can thereby easily be executed, although estimating the volume of the complex geometries is not always feasible. Thus, weight-based swelling ratios are usually reported in the literature [21,22]. However, these methods do not provide insight into the time-dependent, spatially resolved hydration of the inner hydrogel structure and require the full removal of excess liquids without withdrawing liquid from the hydrogel network.

Therefore, an easily transferable, site-resolving, nondestructive 3D analytical method is desired for material development. Magnetic resonance imaging (MRI) is a 3D imaging technique that is used in medical diagnostics, material sciences, and food research [23]. The image contrast thereby often relies on the NMR relaxation properties and spin density differences in a material [24,25,26]. Recently, it was shown to be applicable to the investigation of complex 3D objects obtained by 3D printing [27] and of different common bioink materials [28]. Setting aside the high initial costs and complexity of operation, the main advantage of MRI is its independence from the analyzed material and from optical transparency, enabling volumetric time and spatially resolved longitudinal studies on the same object [23,29]. 

Initially, traditional material characterization of the hydrogel material—based on sodium alginate and dityrosine-crosslinked casein [30]—was performed by swelling studies as well as rheometric analysis and uniaxial compression of casted discs using four different liquid systems. Following this, macroscopic auxetic structuring introduced by 3D printing was used for further functionalization of the protein-based hydrogel. In order to assess the now-complex structure of the hydrogel, we applied MRI, assessing time-dependent external and internal stimuli behavior. As a proof of principle, the 2D-crosssections of an auxetic geometry were analyzed with regard to their changes in the two-dimensional plane. Monitoring included the observation of visual structural changes of the lattice network as well as the estimation of area ratios of the hydrogel cross sections by using image analysis.

## 2. Materials and Methods

### 2.1. Buffer and Stock Solutions

The formulation buffer for material fabrication consisted of 20 mM sodium phosphate buffer (SPB) pH 8 containing 4 M urea. All liquid systems were prepared with ultrapure water (PURELAB Ultra, ELGA LabWater, Lane End, UK). At a temperature of 22 °C, the pH was adjusted using a 4 M sodium hydroxide solution and the buffer filtered through an 0.45 µm membrane (Pall Corporation, New York, NY, USA). The photoinitiator tris(2,2′-bipyridyl)dichlororuthenium(II) hexahydrate (Ru(bpy)_3_Cl_2_·6H_2_O) was diluted in the formulation buffer with a concentration of 5 mM and stored at 4 °C as a stock solution. A 2 M stock solution of the oxidant ammonium persulfate (APS, chemical formula: (NH_4_)_2_S_2_O_8_) was prepared in the formulation buffer, stored as aliquots at −20 °C, and thawed directly prior to use.

To examine swelling or the effect of external stimuli in different systems, four different liquid systems for immersion of the dried hydrogel were prepared. These liquids were as follows:

- System 1: ultrapure water;
- System 2: ultrapure water + 0.1 M CaCl_2_;
- System 3: ultrapure water + 0.1 M CaCl_2_ + 4 M urea;
- System 4: ultrapure water + 20 mM SPB + 4 M urea.

For all liquids, pH (pH Meter HI 3220-2, Hanna Instruments, Woonsocket, RI, USA, equipped with the pH electrode Sentix^®^ 62, Xylem Inc., Rye Brook, NY, USA) and conductivity using a conductivity meter CDM230 (Radiometer Analytica SAS, Lyon, France) were determined (see Supplementary Information S1). All chemicals reported in this manuscript were purchased from Merck KGaA (Darmstadt, Germany).

### 2.2. Ink Preparation

The ink formulation consisted of 10 wt% casein, 0.25 mM Ru(bpy)_3_Cl_2_, 75 mM APS, 3.5 wt% sodium alginate (SA), and the formulation buffer described above. Therefore, SA was dissolved in 60% of the total buffer volume in a dual asymmetric centrifuge (DAC, SpeedMixer^®^ DAC 150.1 FVZ-K, Hauschild GmbH & Co. KG, Hamm, Germany) at 2500 rpm. Subsequently, the remaining formulation buffer, casein powder, and photoinitiator stock solution were added and mixed in the DAC (2500 rpm, 5 min). To enable the induction of the photopolymerization reaction, the precursor solution of the ink was finalized by addition of the APS stock solution directly prior to polymerization using the DAC (2500 rpm, 2 min).

### 2.3. Disc Polymerization

After the addition of APS, the precursor solution of the ink was transferred into a cylindrical polytetrafluoroethylene mold (diameter 10 mm, height 3 mm) that was covered on top and bottom with a transparent hydrophobic layer and acrylic glass. The precursor solution was irradiated for 5 min from above and below using a blue light-emitting diode (LZ4-00B208, LED Engin Inc., San Jose, CA, USA) with a radiant flux of 3.9 W at a distance of 7.5 cm. The discs were released from the mold and dried for 72 h at 40 °C (T6120, Heraeus Holding GmbH, Hanau, Germany).

### 2.4. Extrusion Printing

An auxetic structure was 3D-printed using a BioScaffolder 3.1 (GeSiM mbH, Radeberg, Germany) after adding the APS stock solution. The scaffold design consisted of nine point-symmetrically arranged re-entrant honeycomb unit cells with curved edges and had a total height of 3 mm (see Supplementary Information S2). The printer was equipped with a pneumatic cartridge dispenser (GeSiM mbH), light-blocking cartridges (10 mL, Nordson Corporation, Westlake, OH, USA), and conic ultraviolet light-blocking nozzles (0.41 mm inner diameter, VIEWEG GmbH, Kranzberg, Germany). A velocity of 1.6 mm/s, strand height of 0.1 mm, start pause of 0.3 s, end pause of 0.1 s, and vertical tear off with an applied pressure of 19 kPa were set as printing parameters. Photopolymerization was initiated after each layer by 60 s illumination using the blue light-emitting diode, as described in Section 2.3.

### 2.5. Magnetic Resonance Imaging (MRI)

The 3D-printed auxetic structures were dried for 72 h at 40 °C, and the time-dependent swelling behavior was characterized by MRI using an Avance HD III SWB 200 MHz tomograph (Bruker BioSpin MRI GmbH, Ettlingen, Germany) applying fast low-angle shot (FLASH) pulse sequence (experiment parameters are summarized in Supplementary Information S3). To speed up the measurement time for a meaningful time resolution, a compromise between resolution and measurement time was found by measuring a stack of 2D measurements (referred to as 2.5 D MRI). FLASH was chosen as a fast and reliable pulse sequence that allows contrast generation mainly along T1 and T2*. The image contrast was found to be adequate for the purpose of the manuscript after optimization of the MRI parameters for the investigated samples.

A total number of 10 slices along the z axis without a distance between the cross sections were simultaneously imaged. Each slice summarizes the signal of a *z*-axial distance of 0.6 mm. In all cases, the hydrogel specimens were placed in a flat-bottom glass vial (25 mm diameter) containing 3 mL of the respective liquid system. Subsequently, the glass vial was manually positioned into the magnet, and correct sample positioning was tested by an initial measurement (<10 s). The measurement sequence (*t*_0_–*t*_17_) was initiated 60–120 s after liquid addition, with the first measurement being referred to as *t*_0_. The time series consisted of 18 measurements with a time interval of 1.7 min each.

To optimize the liquid-to-hydrogel contrast, hydrogel specimens immersed in 0.1 M CaCl_2_ within the magnet were additionally measured by applying rapid acquisition with relaxation enhancement (RARE) pulse sequences with the same time interval (experimental parameters are summarized in Supplementary Information S4) in the images. Due to the slightly different chemical composition, this step was essential for being able to apply the subsequent data analysis steps with high enough reliability. The chosen MRI parameters provide in this case a combined weighting by T1 and T2, while the T1 weighting is reduced compared to the FLASH parameters. For the numerical details, see Table S3. In all cases, the ^1^H spin signal of all 10 slices was transferred into a grayscale representation for visualization purposes.

### 2.6. Image Analysis

**Figure 1:**
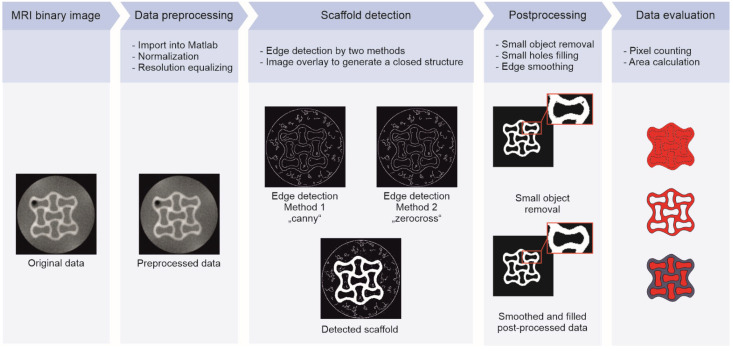
Overview of the image processing. After import into MATLAB^®^, raw image files were preprocessed by normalization and resolution equalization. The scaffold was detected for the fifth slice in the *z*-direction in each data set, whereas small objects were removed during image postprocessing. Scaffold defects were filled and edges smoothed to determine the hydrogel lattice and interstitial area in the measured slice.

MRI data of the fifth slice in the z-direction were processed in MATLAB^®^ R2021b (The MathWorks, Natick, MA, USA). An overview of the image processing is shown in Figure 1. All pixel intensities were normalized to the maximum intensity in the slice. The image resolution was aligned to the highest resolution measured in the data set (function “imresize”, bilinear method) to minimize statistical errors during the following edge detection and overall pixel counting. Due to the small liquid-to-hydrogel contrast in some images, two edge detection methods were performed for each file (function “edge”, methods “canny” and “zerocross”). An overlay of the resulting images was used to generate and fill structures. If necessary, non-continuous edges were manually connected to generate an enclosed area. In subsequent postprocessing, small objects (<2000 px) were removed, small holes (<150 px) in the structure were filled, and the structure edges were smoothed. Finally, the areas of the lattice and interstices were determined by counting the pixels of interest multiplied with the image resolution. The obtained area ratio (AR) is defined as follows:

(1) $$AR_{System,t}=\frac{Area_{System,t}}{Area_{System,\;t_{0}}}$$

where *Area_System,t_* describes the area of either the hydrogel lattice, interstices, or the overall hydrogel area being a combination of the area determined for lattice and interstices (see Figure 1, Data Evaluation) depending on the four liquid phases (System 1 to System 4) at distinctive time points *t* and *t*_0_, respectively (number of replicates (*n*) = 2).

Distances (in the Section 3 and Section 3.4) were estimated with ImageJ V1.53k (NIH, Bethesda, MD, USA) of distinct measurements, and their length ratios (LR) are defined as follows:

(2) $$LR_{System,t}=\frac{Distance_{System,t}}{Distance_{System,\;t_{0}}}$$

where *Distance_System,t_* refers to the length of longitudinal and transverse cross section of the overall structure or the inner unit cell, depending on the four liquid systems at distinctive time points *t* and *t*_0_ (*n* = 2).

### 2.7. Additional Hydrogel Characterization Methods

#### 2.7.1. Weight-Based Swelling Studies

The hydrogel discs were weighted as prepared (m_0_) after being dried for 72 h (m_dry_0_), soaked in liquid (m_1_), and being dried again for 72 h (m_dry_1_). These weights were used to determine the swelling ratio m_rel_ = m_1_/m_0_ as well as the dry-weight ratio m_dry_rel_ = m_dry_1_/m_dry_0_ (*n* = 3).

#### 2.7.2. Oscillatory Rheometry

Prior to analytics, previously dried hydrogel discs (preparation see Section 2.3) were stored for 7 d in each of the four liquid systems (see Section 2.1) with a 100-fold liquid excess for monitoring of the swollen state. Amplitude sweeps (angular frequency ω = 1 and 25 rad·s^−1^, shear stress τ = 5–10.000 Pa, *n* = 2) and frequency sweeps (τ = 10 Pa and ω = 1–25 rad·s^−1^, *n* = 3) were performed on a Physica MCR 301 plate rheometer equipped with a plate–plate geometry (PP10, diameter 10 mm, all Anton Paar GmbH, Graz, Austria) at 22 °C. To reduce the influence of potential outliers, the storage modulus G′ and the loss factor tan δ (=G″/G′ with G″ being the loss modulus) of a hydrogel were determined by averaging all data points obtained by the frequency sweep measurements after excluding outliers, which were identified by a difference of three scaled median absolute deviations from the median.

#### 2.7.3. Uniaxial Compression Tests

Sample preparation for uniaxial compression tests was executed along the lines of the rheometry tests (see Section 2.7.2). Accordingly, hydrogel discs were dried and stored in each liquid system for 7 d prior to analysis of the swollen state. Uniaxial compression tests were performed either on a universal testing machine (zwickiLine Z0.5TN or Z2.5) equipped with either a Xforce HP 100 N (all ZwickRoell GmbH & Co. KG, Ulm, Germany) for forces ≤ 100 N or a KAP-Z 1 kN (Angewandte System Technik GmbH, Dresden, Germany) load cell and stainless-steel compression platen (diameter 30 mm, ZwickRoell GmbH & Co. KG, Ulm, Germany) for forces > 100 N. Initial hydrogel length was determined at a preforce of 0.2 N, with a uniform velocity of 2 mm/min being applied until sample fracture to determine the compressive strength σ_max_ and the fracture strain ε_max_ (*n* = 5).

## 3. Results

### 3.1. Hydrogel Characterization

Initially, commonly applied analytical methods for hydrogel characterization, such as weight-based swelling studies, rheometric analysis, and uniaxial compression, were performed. The results are given in Table 1. The weight-based swelling ratio m_rel_ was found to be dependent on the external stimuli of the different liquid systems applied. Comparable results were obtained for the swelling ratio of the same hydrogel specimens in their dried state m_dry_rel_. The network density, which is correlated to the storage modulus G’, as well as the loss factor tan δ (=G″/G′ with G″ being the loss modulus) to describe the elasticity, were determined by rheometric analysis. The network density was the lowest (32 kPa) for equilibration in formulation buffer (System 4), increasing to 75 kPa for ultrapure water (System 1), and reaching an increased network density of 100 kPa (System 2) and 104 kPa (System 3) for liquids containing CaCl_2_. The elasticity was the highest in formulation buffer, as stated by the lowest tan δ, while decreasing upon CaCl_2_ addition, and a further decrease occurred when urea was removed or even more when only water was used as liquid. The specimen equilibrated in formulation buffer was the least stable, with a fracture strain of 44 ± 9% and a compressive strength of 0.06 MPa. Upon addition of CaCl_2_, these were increased to 73% elongation and 0.78 MPa. The uniaxial compression analysis could not be evaluated with the used analytical setup for liquids without urea, as only partial sample rupture occurred without a clear break point in the stress–strain curve.

### 3.2. Magnetic Resonance Imaging (MRI)

**Figure 2:**
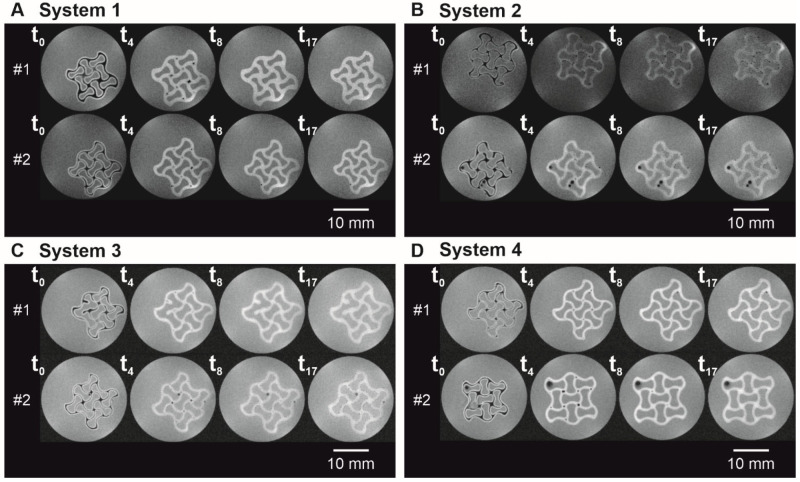
Auxetic structures in different liquid phases in MRI grayscale images with a field of view of 25 × 25 mm² at distinct time points. The liquid phase was (**A**) System 1, (**B**) System 2, (**C**) System 3, and (**D**) System 4 (*n* = 2).

In order to study the liquid immersion into a previously 3D-printed and subsequently dried auxetic hydrogel and thus the swelling behavior, time- and spatially resolved MRI was performed using FLASH pulse sequences. To study the immersion of different liquids in a previously 3D-printed and subsequently dried auxetic hydrogel, time- and spatially resolved MRI was performed. Therefore, the measurement sequence of FLASH pulses was set to a time span of 1.7 min between two measurements. Figure 2A–D display four image series highlighting four exemplary points in time (*t*_0_ = 1.7 min; *t*_4_ = 8.5 min; *t*_8_ = 15.3 min; *t*_17_ = 30.6 min) within a measurement sequence for each condition.

**Figure 3:**
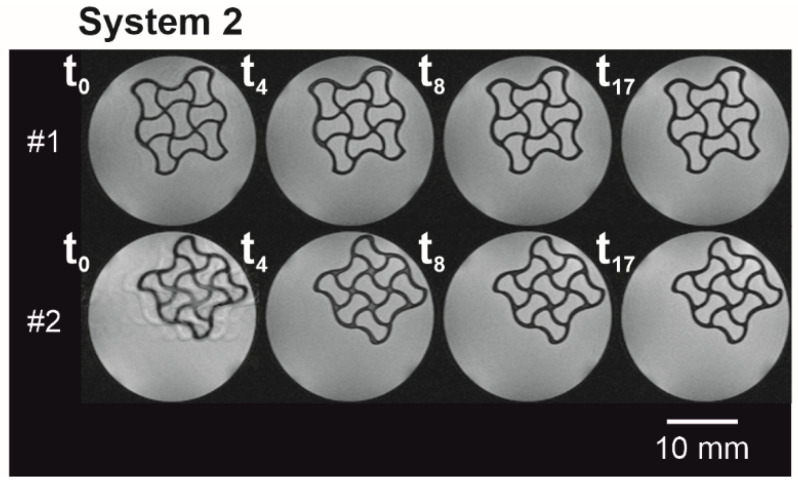
Auxetic structures in System 2 in MRI grayscale images with a field of view of 25 × 25 mm² at distinct time points. The MRI measurements’ settings were optimized towards an increased buffer-to-hydrogel contrast (*n* = 2).

For all conditions, the measurements at t_0_ showed liquid penetration in the outer edges of the hydrogel species (white color), whereas the inner core of the structure was still dry (black color, Figure 2A–D, *t*_0_). With ongoing time, the white area within the hydrogel lattices increased, visualizing the wetting and subsequent swelling of the scaffold. Although the liquid immersed into the hydrogel and was present throughout the strands at *t*_4_ = 8.5 min, there were still dry spots at the nodal points of the hydrogel network observable. Except for two little black spots (Figure 2D, sample #2, *t*_8_), the liquid had penetrated the hydrogel networks completely for *t*_8_ = 15.3 min. When looking (visual inspection) at the experimental data for hydrogel specimens immersed in formulation buffer (Figure 2D) and ultrapure water (Figure 2A), a stimuli-dependent behavior can be seen in the different lattice thicknesses and overall hydrogel dimensions. As such clear changes could not be perceived in systems 2 and 3, an image analysis was made in the following to quantify and visualize changes in the hydrogel dimensions more reliably. The low liquid-to-hydrogel contrast for samples immersed in 0.1 M CaCl_2_ samples (Figure 2B) hindered a meaningful image analysis; therefore, an application-optimized MRI measurement (Figure 3) by RARE pulse sequences was performed. For visualization purposes, the colors in these images were swapped, meaning that black corresponds to a high signal and white to a lower signal. Due to movement of the sample within the liquid, the first image of the second replicate showed blurring, as the image generation in MRI integrates the signals over the measurement time—here, 1 min 42 s. Artefacts—most likely air bubbles adhering to the hydrogel scaffold—in the interstices of the structure are found in two time series (sample #2 in Figure 2B,D).

### 3.3. Swelling Behavior—Area Ratios

**Figure 4:**
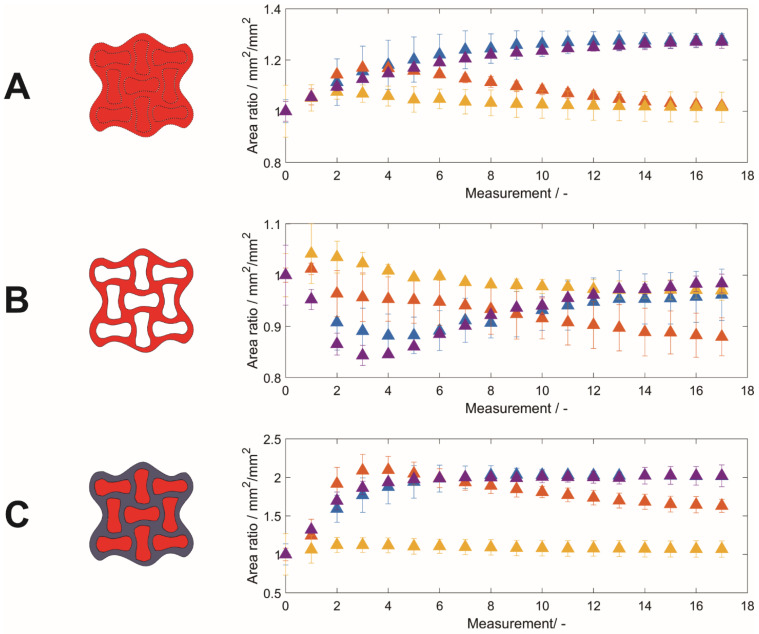
Areas of (**A**) the hydrogel specimen, (**B**) lattice, and (**C**) interstices relative to *t*_0_ depending on the liquid phase as a function of time. The evaluated area is highlighted in the corresponding exemplary structure. Data is expressed as the mean ± empirical standard deviation of two hydrogel specimens per condition.

To quantify the swelling behavior as a function of the liquid used, the hydrogel lattice and the interstices, as well as the area covered by the hydrogel construct, were determined by image analysis (Figure 4A–C). The MRI images of the fifth layer in the z-direction were chosen to ensure comparable image quality. As the overall sample size may vary due to imperfect structures (e.g., by variation during sample processing or printing defects), the area obtained for different time points was normalized to the area obtained at *t*_0_. Previously dried hydrogel specimens placed in urea-containing liquid phases (System 3, System 4) showed an overall increase in their area by about 27%, whereas hydrogel specimens placed in liquid phases without urea (System 1, System 2) started to increase their area by 17% (System 1) or 7% (System 2) and were then shrinking, approximately covering the initial area again. The course of the area ratio according to Equation (1) is shown in Figure 4A.

Having a closer look at the composition of the overall hydrogel dimension, one has to distinguish the covered area by the hydrogel lattice and the enclosed interstices filled with the liquid phase. In contrast to the overall dimension, a different time-dependent trend is found for the lattices: the lattice area decreased by 12 ± 4% (System 4) at *t*_5_ and 16 ± 2% (System 3) at *t*_4_ for urea-containing liquids (Figure 4B). Subsequently, there was a steady increase over the time period considered, almost reaching the initial area for these two conditions. For both liquids without urea (Systems 1 and 2), the area increased for the second time point and then steadily decreased. Systems 2–4, containing salt or salt and urea, leveled approximately at the same area ratio of 0.96 ± 0.05 (System 4) to 0.98 ± 0.02 (System 3), whereas the area ratio of System 1 decreased to 0.88 ± 0.04.

When analyzing the interstices for urea-containing solutions (System 3; System 4), the covered area steadily increased for the first eight time points *t*_0_–*t*_8_ and reached a constant area of approximately twice the area compared to the beginning. For System 2, the interstitial area increased considerably less, with a maximum of 12 ± 10% at *t*_4_ and a slight decrease in interstitial area thereafter. Hydrogel specimens in ultrapure water (System 1) exhibited the smallest geometry changes. Initially, swelling to a maximum area of 109 ± 21% at *t*_4_, followed by a constant decrease without reaching a plateau at *t*_17_, was documented. 

### 3.4. Swelling Behavior—Distances

**Figure 5:**
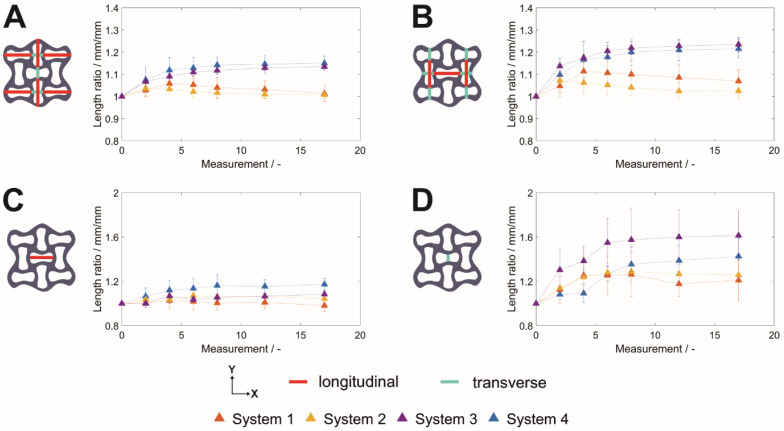
Time-dependent length of structural elements of the auxetic lattice consisting of nine point-symmetrically arranged unit cells. (**A**) Two longitudinal unit cells and one transverse unit cell, (**B**) one longitudinal and two transverse unit cells, and the (**C**) longitudinal as well as (**D**) transverse dimension of the center unit cell. The distances are highlighted in the corresponding exemplary structure. Data are expressed as the mean ± empirical standard deviation of two hydrogel specimens relative to the value at *t*_0_ with one (**C**+**D**) or three lines per sample (**A**+**B**), with two samples per condition.

Auxetic structures are applied in technical settings because of their characteristic anisotropic elongation and shortening under stress. By MRI, this can be measured by observing the time-dependent geometric changes of distances within the nine point-symmetrically arranged re-entrant honeycomb structures. The geometry of the cells of the honeycomb, which can be separated into longitudinal and transverse unit cells, was investigated by comparing the length ratio between longitudinal and transverse unit cells (Equation (2)). As displayed in Figure 5, the x-direction of the outer rows consists of two longitudinal (marked in red) and one transverse (marked in turquoise) unit cell (Figure 5A). In the y-direction, one longitudinal and two transverse cells can be found. Comparing Figure 5A,B, the length ratio was steadily increasing for urea-containing liquids to a maximum of 1.22 (System 4) and 1.23 (System 3) at *t*_17_, whereas the length ratio increased and decreased again to values similar to the starting value for non-urea-containing liquids (System 1; System 2) for both orientations. Considering the eight monitored hydrogel samples stored in different liquids (*n* = 2), a general trend for a higher value of the length ratio could be observed for the target distances, with a higher percentage of transverse cells contributing to the respective elongation. 

In the center unit of the hydrogel lattice (C+D), the geometry changes might only be influenced by the outer cells, as the liquid diffusion into the dried lattice is possible from all outer edges and no anisotropic external stress was applied. Again, the elongation of the transverse cell was more pronounced than the elongation of the longitudinal central cell when compared to distances measured at *t*_0_. However, influenced by the unfavorable resolution for the longitudinal central cell in Figure 5D, the deviation increases. Based on that, the differences between the urea-containing liquid systems (System 3, System 4) might be caused by the low number of samples and may be enhanced by further optimization of the parameters of the MRI for a more detailed evaluation. 

## 4. Discussion

**Magnetic resonance imaging for hydrogel characterization.** Buffer substances such as urea or salts in general can account for more than half of the as-prepared hydrogel dry weight. By using different liquid phases with special characteristics with and without salts and urea being present, commonly applied weight-based swelling ratios are thus inapplicable, as diffusion of buffer substances into or out of the hydrogel network cannot be neglected. In addition, weight-based swelling ratios are mostly determined using simple geometric structures, such as cylinders or cubes. Thus, the analysis of a more complex auxetic structure with interstices filled with liquid causes difficulties in reproducible removal of excess water.

To overcome these disadvantages, MRI was applied as a new tool to describe the time-dependent stimuli and thus swelling behavior caused by liquid diffusion into an auxetic, protein-based hydrogel network. Using this approach, both the liquid front development as such but also the swelling behavior of outer and inner structures could be traced in a time- and spatially resolved way. Please be aware that potential scaffold defects, such as small holes within the structure, are smoothed during image processing, which can lead to misinterpretations of the liquid penetration time and swelling behavior. This is especially important for production processes, which tend to lead to many structural defects. Since no printing defects were observed during visual inspection of the printed hydrogels (see exemplarily Figure S2)—apart from the still visible incomplete strands in Sample #1, illustrated in Figure 2C, and thus the different hydrogel-to-liquid ratio of this sample—this effect was not further considered in the context of this manuscript. Within the scope of this manuscript, it was possible to trace three different liquids with one analysis method, as shown by a high liquid-to-hydrogel contrast in Figure 2 for liquid systems 1, 3, and 4. By changing the MRI pulse sequence, an optimized liquid-to-hydrogel contrast (Figure 3) allowed the monitoring of hydrogel specimens placed in a fourth liquid (System 2) that could not be previously analyzed using the proposed image analysis method. Overall, these results are indicative of a good transferability of the approach used for the screening of new materials. Further, we can envision the adaption of this method to study short-time diffusional effects of core-shell microcapsules, hydrogel shells which exemplarily encapsulate a liquid core containing bioactive molecules. In the area of tissue engineering, the concept could be suitable for the long-term monitoring of hydrogels containing cells [31] or for liquid diffusion monitoring (e.g., vascularization) and swelling behavior of functional hydrogels.

**Stimuli responsiveness of the used protein-based hydrogel formulation.** In order to discuss the stimuli responsiveness of the hydrogel formulations used, several effects which are responsible for the formation and expansion of a hydrogel network have to be considered, taking into account a more detailed study of the influencing parameters during hydrogel formation published previously [15]. The network formation is caused by the chemical crosslinking of casein by the induced photoreaction, so the main proportion of casein is expected to be incorporated into the hydrogel network. In contrast, the used thickening agent, sodium alginate, is soluble in aqueous solutions and thus may diffuse out of the hydrogel network. However, it can be physically crosslinked by ionic interactions in the presence of divalent cations, such as those introduced by 0.1 M CaCl_2_ in the liquid phase. This double crosslinking was verified by an increasing storage modulus in the rheometric analysis, whereas elasticity decreased upon CaCl_2_ addition and was even further decreasing for liquids without urea with an opposite effect for the compressive strength and maximum elongation.

**Figure 6:**
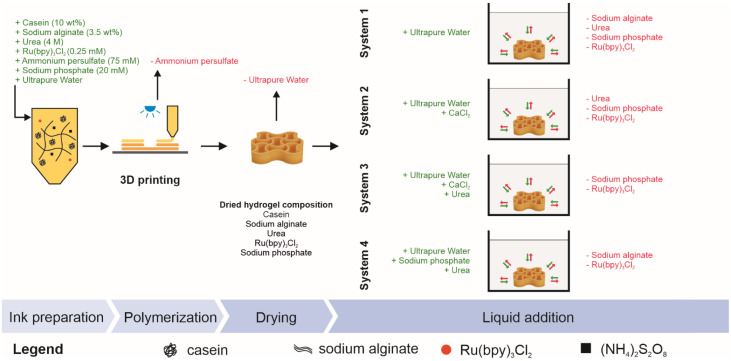
Overview of the chemical substances present in the hydrogel specimens during sample preparation and as a function of the immersion liquid system. Components added to the system (as part of the ink formulation or the different liquid systems used to rehydrate the auxetic structure) are marked in green, whereas components that might be diluted out of the hydrogel specimens are marked in red.

In addition to the formation of the hydrogel network by chemical crosslinking or a combination of the chemical dityrosine crosslinking of casein and physical ionic crosslinking of sodium alginate, interactions inside the hydrogel network are responsible for its expansions and mechanical properties. These interactions are based on a complicated interplay between the crosslinked protein (and sodium alginate) and the formulation buffer components that remain in the (dried) hydrogel specimen, as well as the composition of the immersion liquid system. Concentration differences in the liquid systems and within the hydrogel result in diffusion of the various components into or out of the hydrogel. Figure 6 illustrates the uptake and decrease of chemical substances in the hydrogel specimens, which are either incorporated into the hydrogel network or present in the liquid phase, depending on the immersing liquid. Since the spatially resolved concentration and thus contribution of the individual components to the swelling behavior of the hydrogel were not in the focus of this study and thus not analyzed on a molecular basis, the course of the time-dependent swelling will not be discussed in detail.

In general, an overall volume increase is explained by the liquid immersion into a dry hydrogel network, causing an association of the polymer molecules with water, explaining the initial increase in the overall area for all liquid systems. Thus, intermolecular effects are gaining importance, with increasing liquid content affecting the hydrogel characteristics. The chaotropic agent urea—which is part of the formulation buffer since it enhances the solubility of the hydrophobic protein casein—decreases hydrophobic interactions [32]. Upon drying, together with sodium phosphate as the other formulation buffer substance, it will remain in the hydrogel structure. Therefore, just looking at the urea content in the hydrogel specimens, if no urea is present in the immersing liquid, urea may slowly diffuse out of the hydrogel network. This leads to increasing intermolecular interactions and thus an overall decrease in lattice area and the overall network dimensions. Additionally, due to the presence of salts such as sodium phosphate and CaCl_2_, the ionic strength is altered depending on the liquid system. The higher the ionic strength, which is correlated with the solution’s conductivity, the more ionic interactions cause a volume decrease in hydrogels. Moreover, the pH, and thus the surface net charge of the protein, depends on the used liquid system. For micellar casein with a pI around pH 4.6 [33], a lower protein repulsion is expected in the urea-containing liquid systems (≈pH 8) compared to the non-urea-containing liquids (<pH 7). The combination of these effects results in a complex time and stimuli-dependent behavior of the overall area covered by the hydrogel dimension, as well as the lattice and interstitial area. However, to address the exact reason for the observed time-dependent behavior, the hydrogel structure and its reaction to each stimulus applied should be further understood (e.g., by using advanced material characterization techniques).

**Macroscopic auxetic structuring.** Besides the induction of stimuli-dependent behavior on a micromolecular level, the protein-based hydrogel was functionalized by macroscopic auxetic structuring. This geometric pattern enables site-directed macromolecular responses. Auxetic structures exhibit a negative Poisson’s ratio, meaning that upon an external force in a uniaxial, longitudinal direction (stretching), their perpendicular cross section increases [6], which is opposite to most of the material structures known. As the liquid uptake into a dried structure exhibits a non-uniaxial force, one would not inevitably expect an auxetic behavior upon liquid immersion. However, this was observed upon liquid uptake from the outside, and the cross section as well as the overall dimension increased further for the center unit cell (Figure 5). Dominating re-entrant parts compared to the longitudinal dimension bend the 3D printed object in one direction. Using extrusion-based 3D printing as a fabrication method generated an auxetic architecture with higher strand widths at the nodes compared to the strands, as the material is merging within a printed layer before light-induced crosslinking. The resulting longer diffusion paths at the nodes and the delayed wetting compared to the strands could be visualized in a site-dependent way by MRI. As the directed stretching is more pronounced at the center unit cell of the auxetic structure, which is influenced by diffusion as well as the directed stretching and swelling of the neighboring units, these effects may contribute to and enhance the overall auxetic behavior during liquid uptake.

## 5. Conclusions

We used MRI as a nondestructive tool to investigate the time-dependent and site-resolved liquid immersion into hydrogel as well as their swelling behavior—both depending on the impact of external stimuli. This is especially helpful for the development of functionalized materials with complex geometry—e.g., hydrogels functionalized by macroscopic auxetic structuring through 3D printing—as 4D changes could be monitored within one measurement series. To enhance process understanding of the swelling, which is connected to geometric changes, this method should be applied to different hydrogel constructs as well as more simplified and 3D structures with more replicates per condition to prove statistical relevance of our findings. Within the scope of this manuscript, changes in hydrogel dimension either related to its area or uniaxial length were quantified by image analysis. Edge detection and filtering options allowed us to distinguish the interstices filled with liquid and the immersing liquid front during the liquid uptake, thus capturing the auxetic behavior, which could exemplarily be shown for the core unit cell. As the method is nondestructive, differences in the swelling and deswelling during a time interval of 30 min could be observed for four different liquid compositions, which may also be a favorable material property in some applications. The fast measurement time of MRI allows a site-resolved insight into the hydrogel during liquid uptake. For 3D-printed hydrogels, this strategy could facilitate nondestructive tests and enhanced process understanding in more complex scenarios than the presented study. One potential application could be the site-directed diffusion of small molecules into 3D printed objects, which plays an important role to supply embedded enzymes or cells [34,35]. In material sciences, a detailed data basis regarding time-dependent and stimuli-dependent swelling effects could be obtained even for liquids with high content of salts or other buffer substances that are prone to misinterpretation when solely looking at weight-based swelling ratios. Therefore, we assume MRI to pave the path to enhanced process understanding for determination of the swelling characteristics of hydrogels and especially functionalized hydrogels in materials sciences.

## Figures and Tables

**Table 1:** Swelling ratios and mechanical properties obtained in the swelling studies by oscillatory rheometry and uniaxial compression of hydrogel discs. All measurements were performed at room temperature (22 °C).

Liquid Phase	m_rel_ %	m_dry_rel_ %	G’ kPa	tan δ-	ε_max_ %	σ_max_ MPa
System 1	31.2 ± 1.4	32.9 ± 0.1	75 ± 13	0.22 ± 0.01	- ^a^	- ^a^
System 2	36.0 ± 1.3	36.9 ± 0.3	100 ± 13	0.13 ± 0.01	- ^a^	- ^a^
System 3	59.3 ± 1.4	71.0 ± 0.8	104 ± 18	0.06 ± 0.00	73 ± 5	0.78 ± 0.15
System 4	137.8 ± 2.0	122.3 ± 1.2	32 ± 4	0.02 ± 0.00	44 ± 9	0.06 ± 0.02

^a^ Not analyzable.

## Data Availability

The data presented in this study are available on request from the corresponding author J.H.

## Supplementary Materials

The following supporting information can be downloaded at: https://www.mdpi.com/article/10.3390/polym14225023/s1, Table S1: Conductivity and pH of the liquids used for sample hydration; Figure S1: Auxetic hydrogel scaffold; Table S2: MRI device and experimental MRI parameters for the image acquisition of liquid systems 1 to 4; Table S3: MRI device and experimental MRI parameters for the image generation for the optimized buffer-to-hydrogel contrast for liquid system 2.
