# Supplementary material for: Magnetic Resonance Imaging: Time-Dependent Wetting and Swelling Behavior of an Auxetic Hydrogel Based on Natural Polymers
Source: Polymers (Basel). 2022 Nov 19;14(22):5023. doi: 10.3390/polym14225023 (PMC9694485; doi:10.3390/polym14225023)
Supplement: Supplementary file 1 [file polymers-14-05023-s001.zip › polymers-1974285-supplementary.pdf]

# Supplementary Material

**Sandra Haas[1], Barbara Schmieg[1,2], Paul Wendling[1], Gisela Guthausen[3,4], Jürgen Hubbuch[1,2*]**

1 Institute of Process Engineering in Life Sciences, Section IV: Molecular Separation Engineering, Karlsruhe Institute of Technology (KIT), Fritz-Haber-Weg 2, 76131 Karlsruhe, Germany
2 Institute of Functional Interfaces, Karlsruhe Institute of Technology (KIT), Hermann-von-Helmholtz-Platz 1, 76344 Eggenstein-Leopoldshafen, Germany
3 Institute of Mechanical Process Engineering and Mechanics, Karlsruhe Institute of Technology (KIT), Adenauerring 20b, 76131 Karlsruhe, Germany
4 Engler Bunte Institut, Water Chemistry and Technology, Karlsruhe Institute of Technology (KIT), Adenauerring 20b, 76131 Karlsruhe, Germany

## S1. Liquid characteristics

**Table S1 -** Conductivity and pH of the liquids used for sample hydration (n = 2)

| Buffer | Conductivity / mS/cm | pH / - |
|---|---|---|
| System 1 | $< 1 \cdot 10^{-3}$ | 6.41 ± 0.32 |
| System 2 | 18.4 ± 0.2 | 6.45 ± 0.47 |
| System 3 | 14.9 ± 0.2 | 8.04 ± 0.09 |
| System 4 | 2.9 ± 0.3 | 8.00 ± 0.02 |

## S2. Auxetic hydrogel scaffold

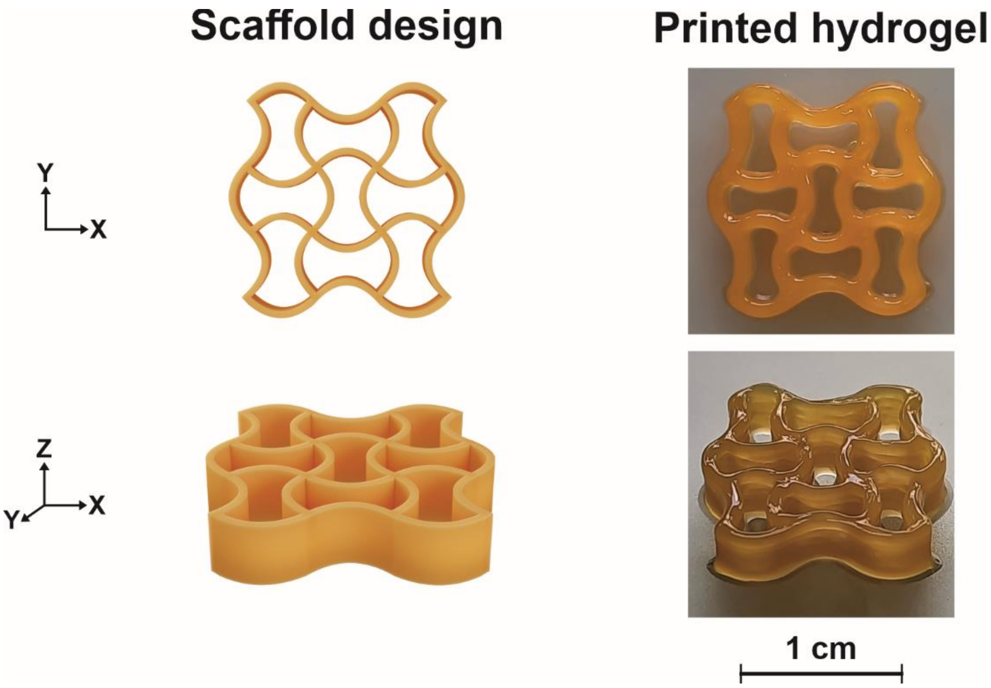


**Figure S1 –** Design of the auxetic scaffold consisting of nine point-symmetrically arranged re-entrant honeycomb structures and one of the 3D printed auxetic protein-based hydrogels before drying

## S3. MRI parameters

**Table S2** – MRI device and experimental MRI parameters for the image acquisition of liquid systems 1 to 4

| MRI parameter | Value |
|---|---|
| MRI probe providing the sample chamber | MIC WB40 RES 200 1H 040/025 QTR |
| Pulse sequence | Fast low-angle shot (FLASH) ortho |
| Field of view | 25 mm x 25 mm |
| Matrix | 128 px x 128 px |
| Number of Slices | 10 |
| Slice thickness | 0.6 mm |
| Slice distance | 0 mm |
| Repetition time | 0.2 s |
| Echo time | 1.88 ms |
| Number of averages | 4 |
| Acquisition time | 1 min 42 s |

## S4. MRI parameters for an optimized buffer-to-hydrogel contrast

**Table S3** – MRI device and experimental MRI parameters for the image generation for the optimized buffer-to-hydrogel contrast for liquid system 2.

| MRI parameter | Value |
|---|---|
| MRI probe providing the sample chamber | MIC WB40 RES 200 1H 040/025 QTR |
| Pulse sequence | Rapid acquisition with relaxation enhancement (RARE) |
| Field of view | 25 mm x 25 mm |
| Matrix | 128 px x 128 px |
| Number of Slices | 10 |
| Slice thickness | 0.6 mm |
| Slice distance | 0 mm |
| Repetition time | 2.13 s |
| Echo time | 10 ms |
| Number of averages | 4 |
| RARE factor | 8 |
| Acquisition time | 1 min 42 s |
